# A comparison of Kato-Katz technique to three other methods for diagnosis of *Amphimerus* spp. liver fluke infection and the prevalence of infection in Chachi Amerindians of Ecuador

**DOI:** 10.1371/journal.pone.0203811

**Published:** 2018-10-04

**Authors:** Manuel Calvopina, Daniel Romero-Alvarez, Fernanda Diaz, William Cevallos, Hiromu Sugiyama

**Affiliations:** 1 OneHealth Group, Carrera de Medicina, Facultad de Ciencias de la Salud, Universidad De Las Américas (UDLA), Quito, Ecuador; 2 Department of Ecology and Evolutionary Biology – Biodiversity Institute, University of Kansas, Lawrence, Kansas, United States of America; 3 Carrera de Medicina, Universidad Central del Ecuador, Quito, Ecuador; 4 Department of Parasitology, National Institute of Infectious Diseases, Tokyo, Japan; George Washington University School of Medicine and Health Sciences, UNITED STATES

## Abstract

**Background:**

Recently, a high prevalence of infection by the liver fluke *Amphimerus* spp. has been documented in the Chachi Amerindians of Ecuador. For diagnosis, no studies exist that compare the sensitivity of different coproparasitological detection techniques. The present study compares the Kato-Katz technique with three other coproparasitological methods for detecting eggs of *Amphimerus* in stools, as well as determines the prevalence of infection in Chachi residents in a Tropical rain forest area in the northwest coast of Ecuador.

**Methodology/Results:**

A total of 105 samples, utilizing the Kato-Katz technique (KK), the spontaneous sedimentation technique in tube (SSTT), the formalin-ether concentration technique (FEC), and direct smear microscopy (DM), were examined. Combining the four methods (fixed “gold” standard), 38 samples were positive with a prevalence of infection of 36.2%. The sensitivities of individual methods were 71%, 58%, 50% and 3% for KK, SSTT, FEC, and DM respectively. Our results indicated that KK alone had the best performance, detecting 27 (71%) of the 38 positive samples. The combination of KK and SSTT detected amphimeriasis in 36 (95%) samples, and KK and FEC in 31 (82%) samples.

**Conclusions:**

DM showed the lowest sensitivity, which raises concern for its value, because it is the standard technique for stool examination for detection of parasites in both public and private laboratories in Ecuador. SSTT alone detected eggs in 22 samples (58%) and would be recommended for field studies because of its simplicity. Performing two techniques on a single sample enhances the detection of *Amphimerus* infection. Its sensitivity is relative to a fixed “gold” standard, determined as the combined results of the four techniques performed. This study confirms the high prevalence of human infection by *Amphimeru*s in the indigenous Chachi group where the first human cases were described.

## Introduction

A high prevalence of human infection by *Amphimerus* spp. liver fluke was recently reported in an indigenous Chachi population located in the province of Esmeraldas, in the northwest tropical region of Ecuador. The prevalence, which varied from 15.5 to 34.1%, was associated with the inland distance from the coast [1]. More recently, a high prevalence of infection in cats and dogs living in the same communities where humans were infected was documented [2]. *Amphimerus* is a genus of trematodes belonging to the Opisthorchiidae family. Other trematodes of this family, *Clonorchis sinensis* and *Opisthorchis* spp., which can also infect the bile ducts, are endemic only in Asian countries [3]. Liver fluke infection is one of the most important food-borne diseases worldwide and is considered by the World Health Organization (WHO) as a neglected tropical disease [4]. *C*. *sinensis* and *Opisthorchis* spp. are classified by the International Agency for Research on Cancer (IARC) as carcinogenic (group 2A and class 1, respectively), considering the high association of their presence and the development of cholangiocarcinoma [3].

The genus *Amphimerus* Barker, 1911 (Trematoda: Opisthorchiidae) has been described mainly in the American continent including Canada, United States, Costa Rica, Panama, Venezuela, Colombia, Ecuador, Brazil, and Peru [1]; it has been also described in South Korea, India and the Philippines [5,6]. *Amphimerus* has not been reported in other geographical regions such as Africa, Oceania, or Europe. Adult worms are hermaphrodites that parasitize the bile ducts and gall bladder, eliminating their eggs via stools, thus, microscopy examination of stool samples searching for eggs is the most commonly used technique for diagnosing *Amphimerus* infection [1,2]. Recently, an ELISA to detect anti-*Amphimerus* IgG in human serum, and LAMPhimerus to detect DNA in feces have been developed [7,8].

In the previous studies of human and domestic animals in Ecuador, diagnosis was made by microscopic observation of *Amphimerus* eggs in stools [1,2]. The only coproparasitological technique used was the formalin-ether concentration (FEC) because the direct smear microscopy (DM) showed a low sensitivity [1]. DM is the only method provided by public and private laboratories for detecting parasite eggs in stools in Ecuador.

WHO recommends the use of the Kato-Katz (KK) method for the detection of human helminth parasites including liver flukes such as *Fasciola hepatica*, *C*. *sinensis* and *Opisthorchis* spp., whose eggs are eliminated via stool. Other methods commonly used include DM, FEC, formalin-ethyl acetate technique, McMaster, FLOTAC and mini-FLOTAC [9]. The method of spontaneous sedimentation technique in tube, (SSTT) as described by Tello in 1988 [10], is a coproparasitological technique with a high sensitivity to detect the majority of intestinal parasites, including eggs, larvae, cysts, and trophozoites, and requires less costly materials and equipment [11–13]. Sedimentation techniques are recommended for general diagnostic laboratories because they are easy to perform and less prone to technical errors [14]. All of these techniques rely on visual examination of prepared stool samples under light microscopy. With the exception of SSTT all of the others use a small amount of fecal material.

For amphimeriasis diagnosis, no studies comparing different coproparasitological detection techniques exist. Thus, the present study was conducted to: (1) determine the best method, or combination of methods to detect the eggs of *Amphimerus* in stool; and (2) to evidence the prevalence of *Amphimerus* infection in Chachi individuals living in a remote tropical village, located in the northwestern coastal region of Ecuador.

## Materials and methods

### Study design and area

This was a cross-sectional study performed in a remote village situated alongside the Cayapas River, in the province of Esmeraldas, latitude of 0.721283°, longitude of -78.906783°, and at an elevation of ~ 34 meters above sea level. The only means of transportation to reach this community is by boat and is located approximately ~135 km inland from the coast. The ecosystem and characteristics of the region has been described in previous studies [1,2,15,16].

### Study population

The study was first introduced and discussed with the local Chachilla in a general assembly, where they were informed about its objectives. Family leaders were asked that all family members participate in the study by providing a stool sample in the next three days. Prospective participants were given a plastic flask for stool collection. All the information was translated into the local language “Chapalache” by a bilingual community health worker. The Chachi indigenous group are Amerindians and represent around 13% of the 24,000 inhabitants of the region. Fishing and farming are their main activities. The total population of the study village was 135 inhabitants.

### Stool collection and parasitological examination

The study was conducted in August 2015. Within a few hours of stool collection, each stool specimen was processed as follows: (1) for DM, a wet mount was prepared with approximately 20 mg of the stool sample suspended in Lugol’s staining solution and subsequently observed microscopically; (2) a single KK thick smear, (3) five grams of fecal material were suspended in 10 mL of warm saline solution for SSTT, and (4) a smear was spotted on a FTA card filter paper, 3 x 2 cm in size and preserved at room temperature for future studies. All samples were processed and observed under light microscopy at a magnification of 100x and 400x, and all results were tabulated while in the community. The remaining amount of fecal material was preserved in 70% ethanol and 10% formalin and transported to the parasitology laboratory in Quito at Centro de Biomedicina, Universidad Central del Ecuador, where it was processed for FEC.

## Coproparasitological techniques

### Kato-Katz (KK)

A 3% methylene blue-glycerol solution was prepared in advance. Cellophane strips of the size of a microscope slide were cut and immersed in the solution for 24 hours before their use. The KK technique was performed following the WHO protocol. A single thick smear slide was prepared using 41.7 mg punched plastic templates. The total *Amphimerus* egg count was recorded and then converted to eggs per gram (EPG) of stool by multiplying the number of eggs per slide by 24 [9].

### Spontaneous sedimentation technique in tube (SSTT)

The protocol used was that described by Tello R. with some modifications [10]. Five grams of fresh feces were weighed and homogenized by strong manual agitation in a 50-mL capped plastic tube containing 25 mL of 0.9% saline (NaCl) solution. It was then filtered through a double-layer surgical gauze, collected in a clean 50-mL plastic tube, filled to 45 mL with warm (40°C) saline, and mixed again for around 30 seconds. If the fecal consistency was hard, it was macerated with a wooden tongue depressor and left upright at room temperature for 2 hours. The supernatant was manually decanted and a sample of the sediment was removed with a plastic pipet. The sample was placed on a glass slide with Lugol´s staining solution, protected with a cover slip, and observed under light microscopy (100x and 400x magnification).

### Formalin-ether concentration (FEC)

The procedure followed was a modified protocol described by Orihel and Ash [17]. Three grams of stool were weighed, homogenized, and diluted to 14 mL with 10% formalin in a 15 mL plastic tube. After being manually shaken, the mixture was filtered throughout a double surgical gauze into a second conical screw capped 15 mL plastic tube. This second tube was allowed to stand for 10 minutes. The supernatant was then manually decanted and the resulting sediment was allowed to stand for 10 minutes. After the addition of 3 mL ethyl ether (Fisher Chemical, New Jersey, USA), the tube was filled to 10 mL with 10% formalin, capped, and manually agitated vigorously for 30 seconds. The sample was centrifuged at room temperature at 2,500 rpm for 5 minutes. This procedure assured the formation of three layers within the tube. The first 2 layers were decanted and then 50 μl of 10% formalin was added to the sediment. Approximately 50 μl of sediment was placed on a slide and concealed with a cover slip. A small drop of Lugol´s staining solution was placed between the slide and cover slip and then examined under a light microscope, scanned first at 100x and then confirmed at 400x magnification.

### Direct smear microscopy (DM)

As previously describe, ~20 mg of fresh fecal material was mixed with Lugol’s staining solution on a glass microscope slide and then examined under a light microscope. Two laboratory technicians observed the samples for the four different methods and then verified by one expert (MC). An individual was considered positive for *Amphimerus* infection if one or more eggs were observed.

### Statistical analyses

Since a referenced “gold” standard test for the detection of *Amphimerus* infection does not exist, the results of the four diagnostic methods employed were used as a fixed “gold” standard to calculate operational characteristics such as the negative predictive value (NPV) [18–20]. The overall reported prevalence of *Amphimerus* infection included all individuals who had at least one test positive for *Amphimerus* eggs. For the comparison analysis, the four individual techniques were used as well as the following six-paired combinations of methods; KK+SSTT, KK+FEC, KK+DM, SSTT+FEC, SSTT+DM, and DM+FC. Data were analyzed using R software (https://www.r-project.org/) to obtain prevalence of infection, sensitivity, and the negative predictive value (NPV) with confidence intervals (CI) calculated at 95%. Concordance between the fixed “gold” standard and individual and paired methods was calculated using the Kappa index. Results were interpreted as of high concordance if Kappa index >0.7 and as of low concordance if Kappa index <0.5; with an *α* = 0.05. Associations between age groups and gender with the fixed “gold” standard were tested using a chi square statistic with an *α* = 0.05. Prevalence and operational characteristics of other soil-transmitted helminths (STH) will be reported elsewhere.

### Ethics

The leaders, the school teachers of the community, and all the individuals involved in the study, signed a participation consent form provided. When children were enrolled, consent was obtained from their guardians. Individuals were free to refuse to participate in the study and provide a stool sample. Ethical committee of the Universidad Central del Ecuador reviewed and approved this study (license number LEC IORG 0001932, FWA 2482, IRB 2483. COBI-AMPH-0064-11). In a second visit to the community, all participants positive for other detected intestinal parasites were treated with antiparasitic medication following the guidelines of the Ecuadorian Ministry of Public Health (MSP).

## Results

A total of 107 individual samples were collected but two were discarded due to inadequate quantity of fecal matter. Thus, all data was analyzed based on 105 samples. Of the participating individuals, 56.2% (59/105) were females and 43.8% (46/105) males, ranging from 1 to 65 years with a mean age of 21.7 years. Age groups were divided as follow: 37% (39/105) belonged to the 1–9 age group, 20% (21/105) to the 10–19 group, 11% (12/105) to 20–29, 12% (13/105) to 30–39, and 19% (20/105) to those more than 40 years. No association was found between age groups, gender, and the prevalence of infection considering the combined results of the four techniques (Table 1).

**Table 1:** Sample population divided by age groups and gender. Prevalence of infection was calculated using the fixed “gold” standard determined as the combined results of all the four diagnostic techniques explored. Percentages were obtained from the number of positives divided by the total number of individuals in each row (Total n).

	Prevalence of infection			Chi Square test	Degrees of Freedom	*p* value
**Age**	*Positive (%)*	*Negative*	**Total n**			
1–9	12 (30.8)	27	39	2.49	4	0.65
10–19	10 (47.6)	11	21			
20–29	3 (25)	9	12			
30–39	5 (30.8)	8	13			
>40	8 (40)	12	20			
Total cases	38 (36.2)	67	105			
**Gender**	*Positive (%)*	*Negative*	**Total n**			
Male	13 (28.3)	33	46	2.23	1	0.14
Female	25(42.4)	34	59			
Total cases	38 (36.2)	67	105			

**Fig 1:**
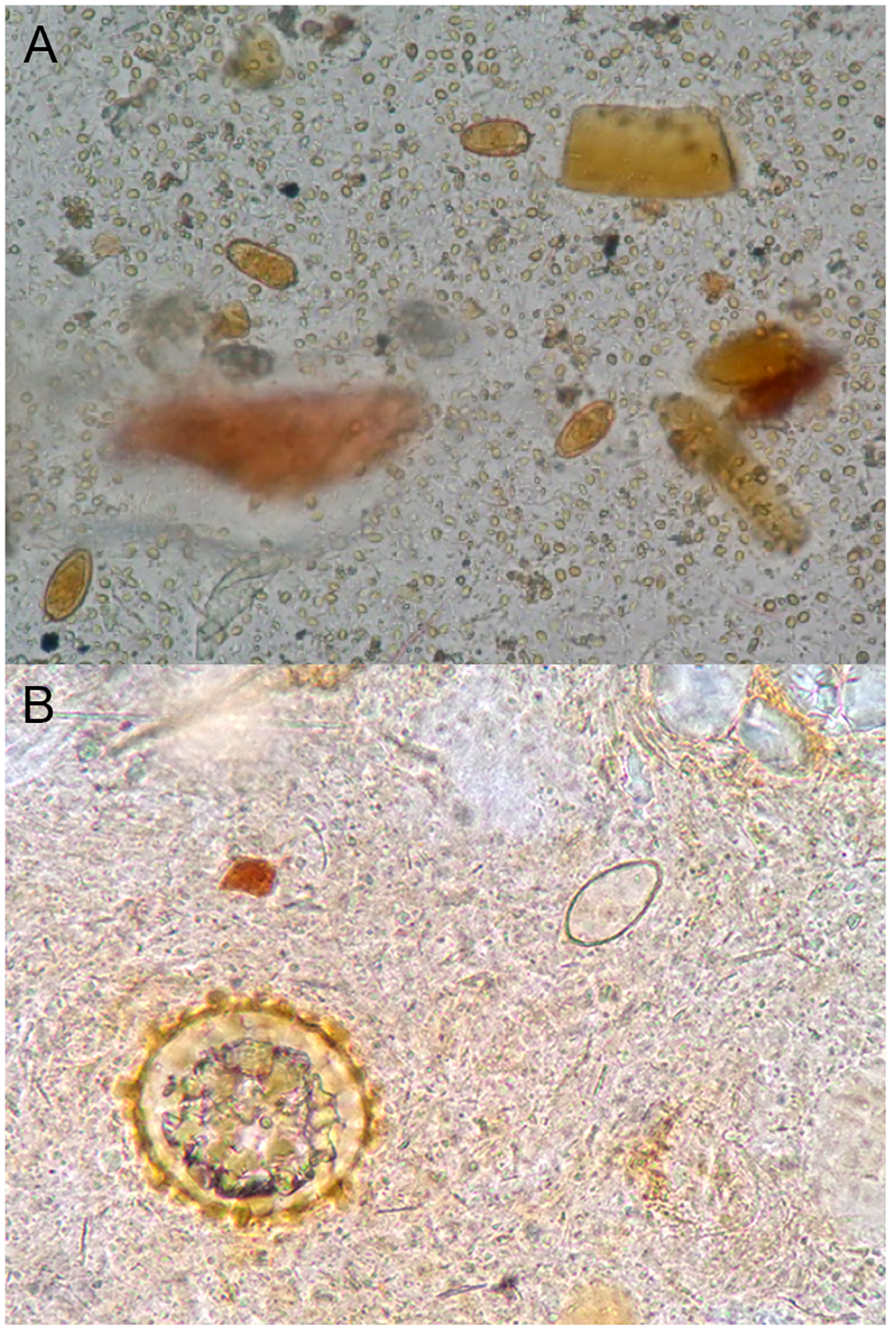
Appearance of *Amphimerus* eggs at different microscopic magnifications and techniques. (A) Four eggs of *Amphimerus* in a Lugol´s stained wet mount (200X magnification). (B) An egg of *Amphimerus* observed in a slide by the Kato-Katz (KK) method (400x) in comparison with a fertile *Ascaris lumbricoides* egg. The eggs observed by light microscopic were oval or piriform-shaped, with a thick yellow-brown shell surrounding it, operculate, measuring 26–33 μm x 13–16 μm, with a small knob seen on the abopercular end, showing the developed miracidium in their interior. In the KK method the interior miracidium disappeared and the membrane became thin and transparent, which present a challenge for egg observation.

Our fixed “gold” standard showed an *Amphimerus* infection prevalence of 36.2% (CI_95%_: 27–46) with 38 of the 105 stool samples positive for *Amphimerus* eggs (Fig 1; Table 1). Individual techniques with the highest *Amphimerus* egg detection ability included KK with 27 (25.7%, CI_95%_: 18–35) and SSTT with 22 (21%, CI_95%_: 14–30) positive samples detected. On the contrary, DM detected only one *Amphimerus* egg (1%, CI_95%_: 0–5; Table 2).

**Table 2:** Prevalence, sensitivity, negative predictive values (NPV) and Kappa index for the individual and combined techniques used in the present study.

		Gold standard (All tests combined)		Overall *Amphimerus* prevalence	CI (95%)	Sensitivity (% positive detected)	CI (95%)	NPV	CI (95%)	Kappa index	Kappa *p* value
**Individual techniques**		*Positive*	*Negative*								
KK	*Positive*	27	0	25.7%	18–35	71%	54–85	86%	76–93	0.76	<0.001
	*Negative*	11	67								
SSTT	*Positive*	22	0	21%	14–30	58%	41–74	81%	71–89	0.64	<0.001
	*Negative*	16	67								
FEC	*Positive*	19	0	18%	11–27	50%	33–67	78%	68–86	0.56	<0.001
	*Negative*	19	67								
DM	*Positive*	1	0	1%	0–5	3%	0–14	64%	54–74	0.33	0.309
	*Negative*	37	67								
**Combined techniques**		*Positive*	*Negative*								
KK+SSTT	*Positive*	36	0	34.3%	25–44	95%	82–99	97%	90–100	0.96	<0.001
	*Negative*	2	67								
KK+FEC	*Positive*	31	0	29.5%	21–39	82%	66–92	91%	81–96	0.85	<0.001
	*Negative*	7	67								
KK+DM	*Positive*	28	0	26.7%	19–36	74%	57–87	87%	77–94	0.78	<0.001
	*Negative*	10	67								
SSTT+FEC	*Positive*	27	0	25.7%	18–35	71%	54–85	86%	76–93	0.76	<0.001
	*Negative*	11	67								
SSTT+DM	*Positive*	22	0	21%	14–30	58%	41–74	81%	71–89	0.64	<0.001
	*Negative*	16	67								
FEC+DM	*Positive*	20	0	19%	12–28	53%	36–69	79%	69–87	0.59	<0.001
	*Negative*	18	67								

The combination of two methods showed a higher sensitivity of detection as compared to any of the individual methods alone. The FEC+DM combination had the lowest sensitivity (53%, CI_95%_: 36–69), while the KK+SSTT methods detected 36 (34.3%, CI_95%_: 25–44) samples reaching a sensitivity of 95% (CI_95%_: 82–99) and a NPV of 97% (CI_95%_: 90–100). The combination of KK+FEC had a sensitivity of 82% (CI_95%_: 66–92) and a NPV of 91% (CI_95%_: 81–96; Table 2).

Concordance between individual and combined tests against our “gold” standard remained equal or above the threshold of low concordance, that is, all analyses showed a Kappa index above 0.5 (*p* <0.001) except for DM with a low concordance (Kappa index = 0.33, *p* = 0.309) (Table 2).

## Discussion

This is the first study to report the sensitivity of KK stool examination technique on *Amphimerus* egg detection in comparison with three other coproparasitological methods and six-paired combinations. Our results demonstrated that the non-duplicated KK method showed the highest sensitivity, identifying 27 (71%) of the 38 stool samples which had been confirmed as *Amphimerus* positive by the four and combined methods. For the second and third ranking, SSTT and FEC found 22 (58%) and 19 (50%) of the 38 positive samples, respectively. Noteworthy, only 1 sample was detected as positive by DM (3%) with a NPV of 64% (Table 2), which is of concern, particularly in Ecuador where DM is the only method provided by private and public laboratories of the MSP.

Currently, there is no “gold” standard diagnosis test for *Amphimerus* liver fluke, even though a recent research effort by our group developed ELISA and LAMP methods with a sensitivity of 85% and 76.6%, and a specificity of 71% and 80.7%, respectively [7,8]. For intestinal parasites, it is widely acknowledged that the analysis of a single stool sample by only one technique can result in a considerable number of false-negative results [21]. Therefore, we used the combined results of 4 different methods as the diagnostic “gold” standard for *Amphimerus* infection as has been used in previous STH studies [20–23]. Combining the results of the 4 methods employed, a prevalence of 36.2% was found for *Amphimerus* infection in the Chachi community studied. The combination of the KK and SSTT results increased the sensitivity to 95% (36/38 positive samples) and by combining KK and FEC methods, a sensitivity of 82% (31/38 positive samples) was obtained. The high NPV of KK+SSTT techniques (97%) add evidence to the high rates of false-negatives when a single stool examination is performed [21]. Both techniques could be implemented to assure the absence of *Amphimerus* in suspected localities. Accordingly, performing 2 techniques on a single sample enhanced the detection of *Amphimerus* infection. However, the results presented should be interpreted considering that the operational characteristics were constrained to the limitations of our sample size (n = 105) and the nature of our selected “gold” standard, which due to its dependence on the other diagnostic tests (i.e., KK, SSTT, FEC, and DM) limited the development of further statistical approaches to support one of the individual or combined techniques over the other among those explored in the present study.

The Kappa index value of 0.33 for DM in this study represents a low concordance of detection in comparison with our fixed “gold” standard, which further substantiates the poor validity of DM. A low sensitivity of DM has been reported for STH infections [3,9,13,20,24], as well as for *Amphimerus* detection [1]. Unlike for KK, SSTT, and FEC, a very small amount of fecal material is processed for DM, approximately 20 mg, which could explain, in part, its low sensitivity. Given the low sensitivity of DM and the high probability of missing *Amphimerus* infections, the employment of this technique should be discouraged for amphimeriasis diagnosis, especially when used alone.

Examination of duplicate 41.7 mg stool samples and/or multiple KK thick smears from one or 2 stool samples and in different days, could enhance the sensitivity of *Amphimerus* diagnosis, but it would increase labor, costs, and be more time consuming [20,23]. Furthermore, processing multiple samples using KK under fieldwork conditions, with limited resources such as power supply, as we did in the present study, would be challenging. It is noteworthy to mention that using the KK method, *Amphimerus* eggs were not detected in 11/38 (29%) stool specimens that were observed using the other 3 methods. This can be explained because during the process the *Amphimerus* eggs can became transparent and thin shelled as is shown in Fig 1 (panel B), and therefore easily missed with the further risk of low detections. They can even disappear due to glycerin when long delays occur between the preparation and microscopic observation. A previous study showed that using duplicate slides for KK, the sensitivity for detecting STH increased from 74 to 95% with high infection intensity; however, dropped to 53 and 80% with low infection intensity [25].

The present study showed that SSTT and FEC methods detected 22 (58%) and 19 (50%) of the 38 positive samples, respectively (Table 2). However, SSTT and FEC detected 9 and 4 extra positive samples respectively that were negative with KK, enhancing amphimeriasis diagnosis. These results might be related to the overall quantity of feces used for each particular technique. SSTT and FEC techniques use 5 and 3 g of fecal matter respectively while only 41.7 mg are used for KK. The SSTT technique also has the advantage of requiring less expensive reagents; just warm saline and two 50-mL capped plastic tubes that could be recycled after careful washing. On the contrary, KK requires 3% malachite-green glycerol or 3% methylene blue-glycerol solution, and FEC method requires 10% buffered-formalin, ether, and centrifuge equipment. SSTT method had been used in several studies for diagnosis of intestinal parasites in developing countries and showed to be highly effective and inexpensive [13]. For this study KK kits were purchased for $400 USD in anticipation of doing 400 stool examinations. According to Tello et al. the cost for SSTT is approximately $0.03 USD per sample [13]. Thus, based on our field experience for detecting *Amphimerus* infection, we would recommend SSTT method because of its simplicity of performing in field conditions, its cost-effective benefits, and its capacity for high volume screening of large populations.

Methods requiring a centrifuge such as FLOTAC have distinct disadvantages in field laboratory settings and also involve more procedural steps. The Mini-FLOTAC needs a closed chamber for flotation and mixing, and a separate reading disc, all resulting in high costs and lengthy procedures. McMaster method is a flotation technique and therefore does not detect trematode eggs such as those of *Amphimerus* or *Schistosoma mansoni* [26].

Another important benefit of the SSTT and FEC methods compared to KK in fieldwork settings is that eggs of Opisthorchiidae members are strong and difficult to break. Therefore, a stool sample can be fixed, transported, and preserved in 10% formalin, merthiolate-iodine-formalin (MIF) or sodium acetate-acetic acid-formalin (SAF) solutions for several days before SSTT and FEC techniques can be performed. Meanwhile, for the KK examination method, stool needs to be processed while fresh and preferably observed the same day. However, a disadvantage for FEC method is that uses reagents that may be hazardous (e.g., ether is highly flammable) or can cause irritation.

This study confirms the high prevalence of human infection by *Amphimerus* in the indigenous Chachi group where the first human infections were described [1]. The prevalence of infection in the present study (36.2%) is higher compared to the previous reported (24%). In the 2011 study, the higher prevalence was reported in the most remote village which was located ~120 km inland [1]. The prevalence reported in the present studied community at ~135 km inland might suggest that villagers living alongside the upper tributaries of the Cayapas River are more infected.

It is important to note that trematode species-specific diagnosis based on egg morphology poses a problem for liver flukes of the Opisthorchiidae and for minute intestinal flukes of Heterophyidae families [27]. The size and shapes of eggs of the members of the above-mentioned families are similar to *Amphimerus* eggs [1,2]. However, these liver and intestinal flukes have not been reported in humans of the Americas [3]. In addition, definitive diagnosis of eggs found in Ecuador was confirmed to be *Amphimerus* by scanning electronic microscopy (SEM) and examination of adult worms obtained from the bile ducts of humans, cats, and dogs in the same area where this study was performed [1,2].

In conclusion, the routine use of DM in both public and private laboratories in Ecuador as the main parasitological diagnostic tool will underestimate the prevalence of *Amphimerus* infections in endemic areas. Its use will also impair the determination of the real geographical distribution of the disease. The use of additional diagnostic methods should be mandatory. The KK technique detected the greatest number of positive samples for eggs of *Amphimerus*. However, 11 (29%) additional samples were detected positive by SSTT and FEC that were missed by the KK method. Our results indicate that SSTT would be an appropriate method in field conditions. The combination of KK and SSTT is likely to detect the majority of *Amphimerus* infections and would be implemented as an effective way to detect trematode eggs in a single stool sample in research or screening settings.

## Supporting information

S1 Dataset Present the structured collected data for age, gender, and positive or negative result for each of the four techniques employed for the 105 samples in this study. (XLSX)
